# Dose-Dependent Effects of Atropine on Accommodative and Binocular Visual Function for Myopia Control in Children: A Systematic Review and Meta-Analysis

**DOI:** 10.1007/s44402-026-00093-5

**Published:** 2026-05-18

**Authors:** Clara Martínez-Pérez, Jacinto Santodomingo-Rubido, César Villa-Collar

**Affiliations:** 1 Applied Physics Department (Optometry Area), Facultade de Óptica e Optometría, Universidade de Santiago de Compostela, Santiago de Compostela, Spain; 2 Global Research and Development, Menicon Co. Ltd, Nagoya, Japan; 3 Faculty of Biomedical and Health Sciences, Universidad Europea, Madrid, Spain

**Keywords:** Accommodation, Axial elongation, Binocularity, Binocular vision, Cycloplegia, Refractive progression

## Abstract

**Purpose:**

To evaluate systematically the effect of different concentrations of atropine eye drops on accommodative amplitude and binocular visual function in children and adolescents with myopia.

**Methods:**

A systematic review and meta-analysis of randomised controlled trials was conducted in accordance with PRISMA 2020 guidelines and registered in PROSPERO (registration number: CRD420261297760). PubMed, Web of Science and Scopus were searched up to January 15, 2025. Eligible studies compared atropine eye drops (0.01–1%) with placebo, single-vision correction or no treatment and reported accommodative or binocular vision outcomes. The primary outcome was the change in accommodative amplitude. Secondary outcomes included accommodative lag, stereoacuity, heterophoria and fusional vergence. Mean differences (MD) with 95% confidence intervals (CI) were pooled using fixed- or random-effects models based on heterogeneity.

**Results:**

Thirteen randomised controlled trials were included, most of which were conducted in Asian populations. Low-dose atropine (0.01%) was associated with a small but statistically significant reduction in accommodative amplitude (MD: −0.84 D, 95% CI: −1.50 to −0.18), with substantial heterogeneity and no consistent effects at individual follow-up time points. Intermediate concentrations (0.02–0.03%) showed variable and heterogeneous effects. Atropine 0.05% produced a consistent and clinically meaningful reduction in accommodative amplitude (MD: −1.96 D, 95% CI: −2.36 to −1.57) and measurable changes in binocular parameters. Higher concentrations (≥0.1%) resulted in marked cycloplegic effects.

**Conclusions:**

The effects of atropine on accommodation and binocular visual function are dose-dependent. Low-dose atropine demonstrates a favourable functional safety profile, while higher concentrations are associated with clinically relevant accommodative impairment.

Key points

- Atropine produces dose-dependent reductions in accommodative amplitude and measurable changes in some binocular vision parameters in myopic children.
- Low-dose atropine (0.01%) shows a generally favourable functional safety profile, with small average changes in accommodation that are often of limited clinical significance.
- Atropine concentrations of 0.05% and higher are associated with clinically meaningful accommodative impairment; clinicians should proactively assess near visual function and consider near additions or other mitigation strategies when symptoms occur.

## Introduction

Myopia has become a major global public health concern due to its increasing prevalence and associated risk of sight-threatening complications later in life [1]. Among the various strategies proposed to slow myopic progression in children, pharmacological, optical and behavioural interventions have been explored [2]. Of these, topical atropine sulphate, a non-selective muscarinic acetylcholine receptor antagonist, has consistently demonstrated strong clinical efficacy in reducing the rate of myopia progression and axial elongation in paediatric populations [2, 3]. Historically, atropine has been widely used in ophthalmic practice for cycloplegia, amblyopia treatment, iridocyclitis and malignant glaucoma [4, 5]. Over the past two decades, this “old drug” has gained renewed interest as a therapeutic option for myopia control following early clinical trials demonstrating that atropine 1% effectively slowed myopic progression [6]. However, the use of high-concentration atropine has been limited due to its association with significant ocular side effects, including prolonged photophobia and near-vision blur, as well as concerns regarding potential systemic adverse effects such as dry mouth, flushing, somnolence, impaired memory and, in rare cases, seizures [7–11].

To mitigate adverse effects while preserving therapeutic efficacy, recent research has shifted toward the use of lower concentrations of atropine. Clinical trials have shown that low-concentration atropine, typically ranging from 0.01% to 0.1%, can effectively slow myopia progression with substantially improved tolerability compared with 1% concentrations [12, 13]. As a result, low-concentration atropine, particularly at the 0.01% concentration, has become the standard of care for myopia control in several Asian countries, with almost half of myopic children in Taiwan receiving atropine eye drops of varying concentrations [14]. Evidence suggests a clear concentration–response relationship, whereby higher atropine concentrations provide greater myopia control but are also associated with more pronounced adverse effects, whereas lower concentrations retain moderate efficacy with fewer side effects [12, 15]. However, the efficacy of 0.01% atropine for myopia control has been increasingly questioned, and recent evidence has contributed to a shift towards 0.05% atropine for progressive myopia in children [16, 17].

Beyond efficacy, a further clinical concern with atropine for myopia control is the rebound acceleration of myopia progression after treatment cessation, which appears to be concentration-dependent. In the Atropine for the Treatment of Myopia (ATOM) studies, rebound was greater following higher concentrations, while pupil size, near visual acuity and accommodation generally recovered after cessation, with quicker recovery at lower concentrations [12]. In ATOM2, higher concentrations provided slightly greater control during treatment but were followed by greater rebound during washout; consequently, 0.01% atropine showed a favourable overall profile at follow-up, combining sustained efficacy with minimal pupil dilation, a modest reduction in accommodation (~2–3 D) and no clinically meaningful near visual loss [12]. These findings have implications for stopping strategies (e.g., tapering rather than abrupt cessation), particularly in younger children who are more likely to require re-treatment. More recently, long-term observational follow-up of ATOM1/ATOM2 participants in the Atropine Treatment Long-Term Assessment Study (ATLAS) reported that short-term atropine exposure (0.01–1.0% for ~2–4 years in childhood) was not associated with differences in adult refractive error or axial length at 10–20 years, although cautious interpretation is warranted given attrition and the observational design [18]. Consistent with the practical challenges of cessation, the 5-year Low-Concentration Atropine for Myopia (LAMP) extended trial reported that most children required restarting atropine after treatment cessation and that re-treatment with 0.05% achieved efficacy comparable to continued therapy, with older age being associated with more successful cessation [17]. Overall, these data reinforce that concentration selection must balance efficacy and tolerability, alongside the likely need for monitoring and planned cessation strategies.

In parallel with these efficacy and safety considerations, understanding atropine’s effects on near visual function is important for optimising clinical prescribing. Despite robust evidence supporting atropine’s efficacy in slowing refractive and axial changes during treatment periods of up to 5 years, the underlying mechanisms by which atropine exerts its anti-myopia effects remain unclear [19]. Traditionally, atropine was thought to control myopia progression primarily by reducing accommodation [2, 3]. However, accumulating evidence suggests that its effects may also involve non-accommodative pathways, including retinal and scleral signalling mediated by muscarinic and adrenergic receptors [20–23]. Given the central role of accommodation and near work in childhood visual behaviour, understanding how atropine influences accommodative and binocular visual function is of particular clinical relevance.

Accommodation plays a critical role in minimising hyperopic defocus during near tasks, and prolonged near work has been consistently associated with myopia development and progression [24, 25]. Previous studies have demonstrated that children with myopia engage in more near work at shorter working distances compared with their emmetropic peers [24]. Alterations in accommodative amplitude, accommodative lag and binocular coordination during near tasks have been hypothesised as potential contributors to myopia progression, as well as potential mediators of atropine-related visual side effects such as near blur and asthenopic symptoms [25]. While several randomised controlled trials have evaluated the effects of atropine on refractive error and axial length, fewer studies have systematically assessed its impact on accommodative and binocular visual function. Reports of adverse binocular outcomes, including reduced accommodative amplitude, changes in heterophoria and isolated cases of convergence excess esotropia following low-concentration atropine use highlight the need for a comprehensive synthesis of available evidence [26–30]. Importantly, it remains unclear whether these effects are concentration-dependent, transient or persistent over time and clinically meaningful in the context of long-term myopia management. Therefore, the aim of this systematic review and meta-analysis is to synthesise evidence from randomised controlled trials (RCTs) to evaluate the effects of atropine eye drops at different concentrations on accommodative amplitude and binocular visual function in children and adolescents with myopia. By examining concentration- and time-dependent effects, this study seeks to clarify the balance between efficacy, visual function and tolerability, thereby providing clinically relevant information to guide evidence-based myopia management.

## Methods

### Research question and PICOS framework

**Fig. 1:**
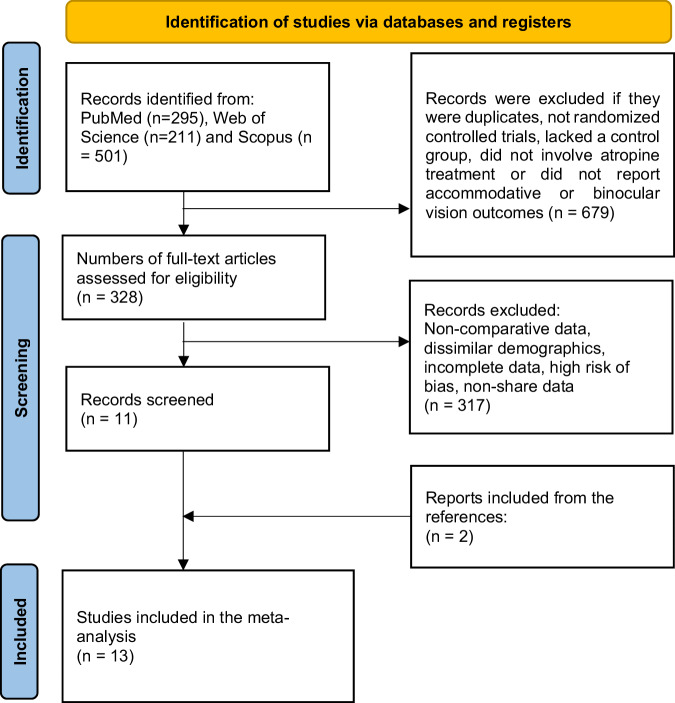
Preferred Reporting Items for Systematic reviews and Meta-Analyses (PRISMA) flow diagram of study selection.

This systematic review and meta-analysis was registered in International Prospective Register of Ongoing Systematic Reviews (PROSPERO) (registration number: CRD420261297760) and conducted in accordance with the Preferred Reporting Items for Systematic reviews and Meta-Analyses (PRISMA) 2020 [31] guidelines and A MeaSurement Tool to Assess Systematic Reviews 2 (AMSTAR-2) methodological standards (Fig. 1). A completed PRISMA checklist is provided as Supplementary Material (Additional file 1). The final literature search was completed on 15 January 2025.

The research question was formulated using the PICOS framework (Population, Intervention, Comparison, Outcomes, and Study design) to ensure methodological rigour and clinical relevance. Specifically, the study aimed to evaluate whether children and adolescents with myopia (Population) experience changes in accommodative and binocular visual function (Outcome) when treated with atropine eye drops at different concentrations (Intervention), compared with placebo, single-vision correction or no atropine treatment (Comparator). Eligible studies were restricted to RCTs (Study design) assessing the effects of atropine on accommodative amplitude and secondary binocular vision outcomes. The primary outcome of interest was the change in accommodative amplitude, assessed using standard clinical methods across different follow-up periods. Secondary outcomes included accommodative lag, stereoacuity, heterophoria (distance and near) and fusional vergence parameters. Differences in atropine concentration and follow-up duration were explored as potential sources of heterogeneity. By synthesising the available evidence, this review clarifies the dose- and time-dependent effects of atropine on accommodative and binocular visual function and provides clinically relevant information to support myopia management decisions and future research.

### Eligibility Criteria

Studies were excluded if they met any of the following criteria: case reports, case series, quasi-experimental designs, cross-sectional studies or uncontrolled trials; systematic or narrative reviews; conference abstracts without full-text availability or duplicate publications derived from the same dataset. Additional exclusions were applied to studies rated as having a high risk of bias or insufficient methodological quality based on predefined assessment criteria, as well as those with non-comparable or incomplete demographic data. Trials were also excluded if they lacked clearly defined diagnostic criteria for myopia, did not include a randomised control group (e.g., placebo or single-vision optical correction) or did not evaluate atropine eye drops as the primary intervention. Studies that failed to report accommodative amplitude as an outcome or did not assess any accommodative or binocular vision parameters were excluded. Furthermore, studies were excluded if they did not provide sufficient statistical information (such as means and standard deviations, confidence intervals or change scores) required for quantitative synthesis in the meta-analysis.

### Information Sources

A comprehensive and systematic literature search was conducted across three major electronic databases: PubMed, Web of Science and Scopus, with no restrictions on publication date or language. The search strategy was designed specifically to identify randomised controlled trials evaluating the effects of atropine eye drops on accommodative and binocular visual function in children and adolescents with myopia. To maximise completeness, the reference lists of all included articles and relevant reviews were also screened manually to identify additional eligible studies not captured through the initial database search.

### Search Methods for Identification of Studies

The search strategy combined controlled vocabulary and free-text terms related to atropine, myopia, accommodative and binocular visual function. Key concepts included the intervention of interest (atropine eye drops), the target population (children and adolescents) and outcomes related to accommodation and binocular vision, such as accommodative amplitude, accommodative lag, stereoacuity, heterophoria, vergence function, near point of convergence and the accommodative convergence to accommodation (AC/A) ratio. Database-specific search strategies were developed and adapted for PubMed, Web of Science and Scopus using appropriate Boolean operators and syntax. Full details of the search strategies applied in each database are provided in Additional file 2. Two reviewers independently screened titles, abstracts and full texts for eligibility, with disagreements resolved through discussion and consensus. No language restrictions were applied. Studies published in languages other than English were translated by the review authors using standard translation tools when sufficient data were available for extraction.

### Data Extraction and Data Items

Two authors independently extracted data from all eligible RCTs. For each study, the following characteristics were recorded: first author, year of publication, country or region, study design, sample size per intervention group, mean participant age, atropine concentration, treatment regimen, duration of follow-up, outcome measures related to accommodative and binocular visual function and disclosure of conflicts of interest. Any discrepancies in data extraction or eligibility assessment were resolved through discussion and consensus. Study management, including duplicate removal and tracking of eligibility decisions, was conducted using Rayyan (Rayyan Systems Inc., rayyan.ai)).

The primary data extracted focused on changes in accommodative amplitude across different follow-up periods. Secondary data included measures of accommodative lag, stereoacuity, heterophoria (distance and near), fusional vergence parameters, near point of convergence and AC/A ratio, when available. Additional information was collected on treatment protocols (e.g., atropine dosage and frequency), study inclusion and exclusion criteria and baseline participant characteristics. These data were used to support subgroup, concentration-based and time-dependent analyses and to explore sources of methodological heterogeneity across studies.

### Methodological Quality and Risk of Bias Assessment

**Fig. 2:**
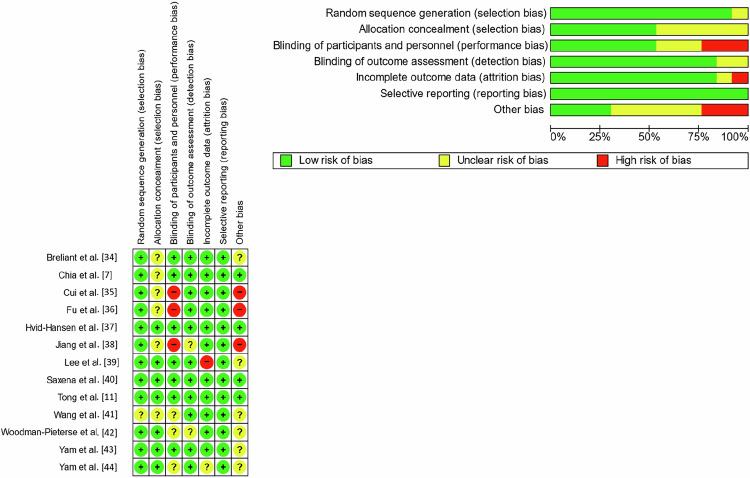
Risk of bias assessment (green = low risk; red = high risk; yellow = unknown) of 13 randomised control trials.

The methodological quality and risk of bias of the included RCTs were evaluated by two reviewers using the Cochrane Collaboration’s Risk of Bias tool, as implemented in Review Manager (RevMan, Version 5.4, The Cochrane Collaboration, cochrane.org). This tool assesses seven key domains of potential bias: random sequence generation, allocation concealment, blinding of participants and personnel (performance bias), blinding of outcome assessment (detection bias), incomplete outcome data (attrition bias), selective reporting (reporting bias) and other potential sources of bias. Each domain was judged as having low, high or unclear risk of bias according to predefined criteria. Disagreements between reviewers were resolved through discussion and consensus. The overall results of the risk of bias assessment for studies evaluating the effects of atropine on accommodative and binocular visual outcomes are summarised in Fig. 2, with detailed domain-specific justifications provided in Additional File 3.

### Publication Bias

Potential publication bias was assessed by visual inspection of funnel plots generated using Review Manager for the primary outcome of accommodative amplitude. Funnel plot asymmetry was interpreted as a possible indication of publication bias or small-study effects, particularly in analyses with a limited number of contributing studies.

### Assessment of Results and Statistical Analyses

For continuous outcomes measured on the same scale, mean differences (MD) with corresponding 95% confidence intervals (CI) were calculated. As accommodative amplitude and other binocular vision parameters were consistently reported using comparable clinical units (e.g., dioptres or prism dioptres (Δ)), no standardisation across measurement scales was required.

Statistical heterogeneity among studies was assessed using the *I*² statistic and interpreted as low (<25%), moderate (25–50%) or high (>50%). A fixed-effects model was applied when heterogeneity was low or not significant (*I*² ≤ 50%), whereas a random-effects model was used in cases of moderate or high heterogeneity.

Sensitivity analyses were conducted to evaluate the robustness of pooled estimates by sequentially removing individual studies identified as major contributors to heterogeneity, particularly for accommodative amplitude outcomes. This approach allowed assessment of the influence of individual trials across follow-up periods and atropine concentrations.

When necessary, missing or incomplete data were handled according to guidance from the Cochrane Handbook for Systematic Reviews of Interventions [32]. All meta-analyses, sensitivity analyses and figure generation were performed using Review Manager.

The certainty of evidence for each atropine concentration was assessed using the Grading of Recommendations, Assessment, Development and Evaluation (GRADE) approach [33], considering risk of bias, inconsistency, indirectness, imprecision and potential publication bias. All assessments were conducted independently by two reviewers, with discrepancies resolved through discussion and consensus.

## Results

### Study Selection

A total of 1007 records were identified through database searching, including PubMed (*n *= 295), Web of Science (*n* = 211) and Scopus (*n* = 501) (Fig. 1). After removal of duplicates, the remaining records were screened by title and abstract and 679 records were excluded because they were not RCTs, lacked a control group or did not report accommodative or binocular vision outcomes. Subsequently, 328 full-text articles were assessed for eligibility. Of these, 317 records were excluded due to non-comparative data, dissimilar demographics, incomplete data, high risk of bias or lack of sufficient data for quantitative synthesis. Two additional studies were identified through manual screening of reference lists. In total, 13 studies met the inclusion criteria and were included in the meta-analysis (Table 1) [8, 11, 34–44].

**Table 1:** Baseline characteristics of the 13 included studies.

Author	Country	Study design	Sample size (*n*)	Mean age ± SD (years)	Atropine (concentration/regimen)	Follow-up	Measures assessed	COI
Breliant et al. [34]	USA	RCT	46	10.7 ± 3.0	Atropine 0.01%, 0.03%, 0.05% vs. placebo (single dose, bilateral)	24 h	Visual acuity (distance, near); pupil size (photopic, scotopic); accommodative amplitude (subjective pull-away method, Bernell rule (Bernell Corporation; bernell.com) [subjective]; accommodative lag (objective open-field autorefraction, Grand Seiko WAM-5500 (Grand Seiko Co., grandseiko.com) at 33 cm) [objective]; heterophoria (distance, near); NPC (break, recovery, stamina, fragility); NFV/PFV	No
Chia et al. [7]	Singapore	RCT	400	NR (6–12)	Atropine 0.5%, 0.1%, 0.01% once nightly (bilateral)	24 months	Myopia progression (cycloplegic autorefraction); axial length (optical biometry); accommodative amplitude (subjective push-up method using RAF near point rule) [subjective]; pupil diameter (photopic, mesopic); visual acuity (distance, near).	No
Cui et al. [35]	China	RCT	400	9.6 ± 1.8	Atropine 0.02% or 0.01% once nightly (bilateral) vs. single-vision spectacles	24 months	Myopia progression (SE, cycloplegic autorefraction); axial length (optical biometry); pupil diameter; accommodative amplitude (subjective push-up method) [subjective].	No
Fu et al. [36]	China	RCT	400	9.4 ± 1.8	Atropine 0.02% or 0.01% once nightly (bilateral) vs. single-vision spectacles	12 months	Myopia progression (SE, cycloplegic autorefraction); axial length (optical biometry); pupil diameter; accommodative amplitude (subjective push-up method) [subjective].	No
Hvid-Hansen et al. [37]	Denmark	RCT	97	9.4 ± 1.7	Atropine 0.1% loading dose (6 months) → 0.01% (bilateral) vs. 0.01% atropine vs. placebo (once nightly)	6 months	Myopia progression (SE, cycloplegic autorefraction); axial length (optical biometry); pupil diameter (photopic, mesopic); accommodative amplitude (RAF rule, push-up/push-down) [subjective]; visual acuity; IOP; adverse events	No
Jiang et al. (2023) [38]	China	RCT	62	10.3 ± 1.1	Atropine 0.01% once nightly (bilateral), alone or combined with OK vs. OK alone vs. placebo spectacles	3 months	Accommodative amplitude (minus-lens method) [subjective]; accommodative lag (MEM) [objective]; NRA/PRA; accommodative facility (MAF, BAF, ±2.00 D flipper); horizontal heterophoria (distance, near, von Graefe); fusional vergence (Risley prism); AC/A ratio (calculated method)	No
Lee et al. [39]	Australia	RCT	153	11.5 ± 2.7	Atropine 0.01% nightly, bilateral vs. placebo	24 months	SE (cycloplegic autorefraction); axial length (optical biometry); accommodative amplitude (RAF rule) [subjective]; pupillary light response (digital pupillometry); best-corrected visual acuity (log MAR); ocular biometry (ACD, lens thickness, corneal parameters).	No
Saxena et al. [40]	India	RCT	100	10.7 ± 2.2	Atropine 0.01% once nightly, bilateral vs. placebo	12 months	SE (cycloplegic refraction); axial length (optical biometry); accommodative amplitude (RAF rule, NPA-derived) [subjective]; pupil size (photopic/mesopic); BCVA; ocular biometry (ACD, LT, VCD)	No
Tong et al. [11]	Singapore	RCT	400	NR (6–12)	No treatment during year 3; follow-up after cessation of prior atropine 1% vs. placebo (uniocular treatment during years 1–2)	36 months	SE (cycloplegic autorefraction); axial length (A-scan ultrasonography); accommodative amplitude (near point of accommodation measured with RAF rule) [subjective]; near visual acuity (logMAR, ETDRS-based chart); ocular biometry (ACD, LT, VCD).	No
Wang et al. (2020) [41]	China	RCT	63	8.7	Atropine 0.01% once nightly + single-vision lenses vs. single-vision lenses	6 months	Accommodation amplitude (monocular and binocular; minus lens method) [subjective]; accommodative facility (±2.00 D flipper); pupil diameter (autorefractor-based); distance and near BCVA (logMAR); near stereoacuity (random-dot stereogram); IOP (non-contact tonometry); SE (cycloplegic autorefraction and retinoscopy); axial length (optical biometry).	No
Woodman-Pieterse et al. [42]	Australia	RCT	20	22.3 ± 1.0	Atropine 0.05% vs. placebo (0.9% NaCl)	10 days	Clinical binocular vision assessment including accommodative amplitude (push-up method) [subjective]; accommodative facility (±2.00 D flipper); relative accommodation (PRA/NRA); accommodative posture (open-field autorefractor, WAM-5500) [objective]; heterophoria (Howell-Dwyer (Cyclopean Design); fusional vergences (PFV/NFV, prism bar); near point of convergence (NPC) and AC/A ratio (gradient method).	No
Yam et al. [43]	Hong Kong (China)	RCT	438	8.4 ± 1.8	Atropine eye drops 0.05%, 0.025%, 0.01% vs. placebo (0.9% NaCl)	12 months	SE (cycloplegic refraction); axial length (IOL Master (Carl Zeiss Meditec AG, zeiss.com)); accommodative amplitude (RAF rule) [subjective]; pupil diameter (OPD-Scan III (Nidek Co., Ltd., nidek.com); best-corrected visual acuity (logMAR)	No
Yam et al. [44]	Hong Kong (China)	RCT	350	8.4 ± 1.8	Atropine 0.05%, 0.025%, 0.01%: continued treatment vs. washout	36 months	SE (cycloplegic refraction); axial length (IOL Master); accommodative amplitude (RAF rule) [subjective]; pupil diameter (OPD-Scan III); best-corrected visual acuity (logMAR)	No

*AA* accommodative amplitude, *AC/A ratio* accommodative convergence to accommodation ratio, *ACD* anterior chamber depth, *BAF* binocular accommodative facility, *BCVA* best-corrected visual acuity, *COI* conflict of interest, *ETDRS* Early Treatment of Diabetic Retinopathy Study, *IOP* intraocular pressure, *logMAR* logarithm of the minimum angle of resolution, *LT* lens thickness, *MAF* monocular accommodative facility, *MEM* monocular estimation method, *n* number of participants, *NaCl* sodium chloride, *NFV* negative fusional vergence, *NPC* near point of convergence, *NPA* near point of accommodation, *NR* not reported, *NRA* negative relative accommodation, *OPD-Scan III* Optical Path Difference Scan III aberrometer/topographer, *OK* orthokeratology, *PFV* positive fusional vergence, *PRA* positive relative accommodation, *RAF rule* Royal Air Force near point rule, *RCT* randomised controlled trial, *SE* spherical equivalent, *SER* spherical equivalent refraction, *VCD* vitreous chamber depth.

### Study Characteristics

Table 1 summarises the characteristics of the RCTs included in this meta-analysis evaluating the effects of atropine on accommodative and binocular visual function. Most trials were conducted in Asian populations, particularly in China, Hong Kong, Singapore and India. Only a limited number of studies originated from Europe, Australia and the United States. Sample sizes ranged from 20 to 438 participants, with most studies involving children aged 8–12 years, although one trial included young adults. All studies employed a randomised controlled design comparing atropine at concentrations ranging from 0.01% to 0.5% with placebo, single-vision correction or alternative treatment modalities. Atropine was generally administered bilaterally once nightly, with follow-up periods varying from short-term assessments (24 h to 10 days) to long-term follow-up of up to 36 months.

Outcome measures primarily focused on accommodative and binocular vision parameters, including accommodative amplitude, accommodative lag, accommodative facility, heterophoria, vergence function, near point of convergence and AC/A ratio. The assessment of accommodative amplitude varied across studies. Most trials used subjective clinical methods such as the push-up technique (e.g., RAF rule) or minus lens methods, whereas a smaller number employed objective measurements using autorefractors or wavefront aberrometers. This variability in assessment methods may contribute to differences in reported accommodative amplitudes across studies. Pupil diameter, visual acuity and ocular biometric variables were also assessed frequently. None of the included studies reported relevant conflicts of interest.

### Outcomes

#### 0.01% Atropine

**Fig. 3:**
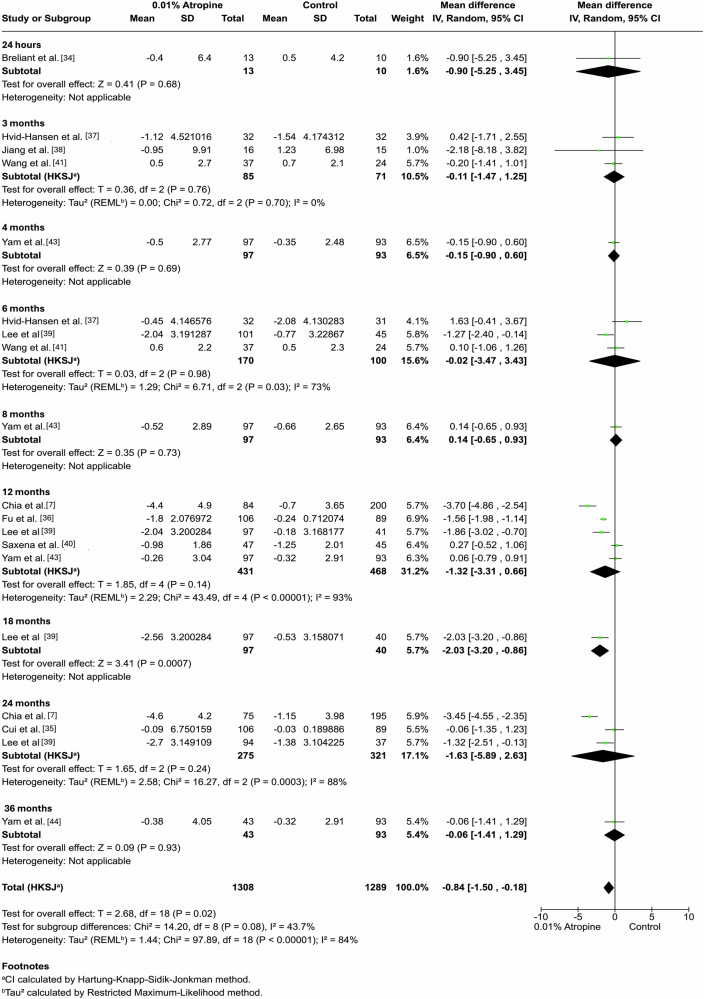
Effect of 0.01% atropine on accommodative amplitude compared with control at different follow-up periods. IV inverse variance, CI confidence interval, SD standard deviation.

Figure 3 summarises the pooled effects of 0.01% atropine compared with control on accommodative amplitude across multiple follow-up periods. No statistically significant pooled differences were observed at early time points (24 h, 3 and 4 months) or at longer follow-up (36 months). At 6 and 12 months, the pooled estimates were not statistically significant; however, substantial heterogeneity was observed, with individual studies reporting divergent effects. A statistically significant reduction in accommodative amplitude was identified at 18 months based on a single study. In the overall analysis, 0.01% atropine was associated with a small but statistically significant reduction in accommodative amplitude compared with control (MD = −0.84 D, 95% CI: −1.50 to −0.18), accompanied by considerable between-study heterogeneity and evidence of temporal variability in treatment effects.

For stereoacuity, no statistically significant differences were observed between the 0.01% atropine and control groups at any follow-up time point. At 3 and 6 months, Wang et al. [41] showed no significant effect of 0.01% atropine on stereoacuity compared with control. Similarly, at 24 months, Lee et al. [39] did not demonstrate significant between-group differences. The pooled analysis across these follow-up periods confirmed the absence of a significant effect of low-concentration atropine on stereoacuity (MD = −0.12, 95% CI: −0.78 to 0.54), with no evidence of heterogeneity.

Regarding accommodative lag, no significant differences were detected between the 0.01% atropine and control groups at either short-term time point. At 24 h, Breliant et al. [34] reported no significant change in accommodative lag following administration of atropine compared with control. Likewise, at 3 months, findings from Jiang et al. [38] showed no significant between-group differences. The overall pooled estimate indicated no significant effect of 0.01% atropine on accommodative lag (MD = −0.15, 95% CI: −0.63 to 0.33), with no heterogeneity across studies.

#### 0.02–0.03% Atropine

For atropine concentrations ranging from 0.02% to 0.025%, several follow-up periods were available. At 4 months, Yam et al. [43] reported a statistically significant reduction in accommodative amplitude in the atropine group compared with the control. At 8 months, no statistically significant differences were observed in the same cohort. At 12 months, pooled data from Fu et al. [36] and Yam et al. [43] showed a reduction in accommodative amplitude in the atropine group; however, the overall pooled estimate did not reach statistical significance (MD = −1.61 D, 95% CI: −3.27 to 0.06). At 24 months, results from Cui et al. [35] demonstrated a small but statistically significant reduction in accommodative amplitude associated with atropine use. At 36 months, findings from Yam et al. [44] did not demonstrate a statistically significant between-group difference.

Overall, the pooled analysis across all follow-up periods indicated that atropine concentrations of 0.02–0.025% were associated with a small but statistically significant reduction in accommodative amplitude compared with control (MD = −0.92 D, 95% CI: −1.57 to −0.28), although substantial heterogeneity was observed across studies.

For 0.03% concentrations, accommodative amplitude did not differ significantly between the atropine and control groups at short-term follow-up. At 24 h, data from a single study by Breliant et al. [34] showed no significant effect of 0.03% atropine on accommodative amplitude compared with control (MD = −1.20 D, 95% CI: −4.38 to 1.98).

#### 0.05% Atropine

**Fig. 4:**
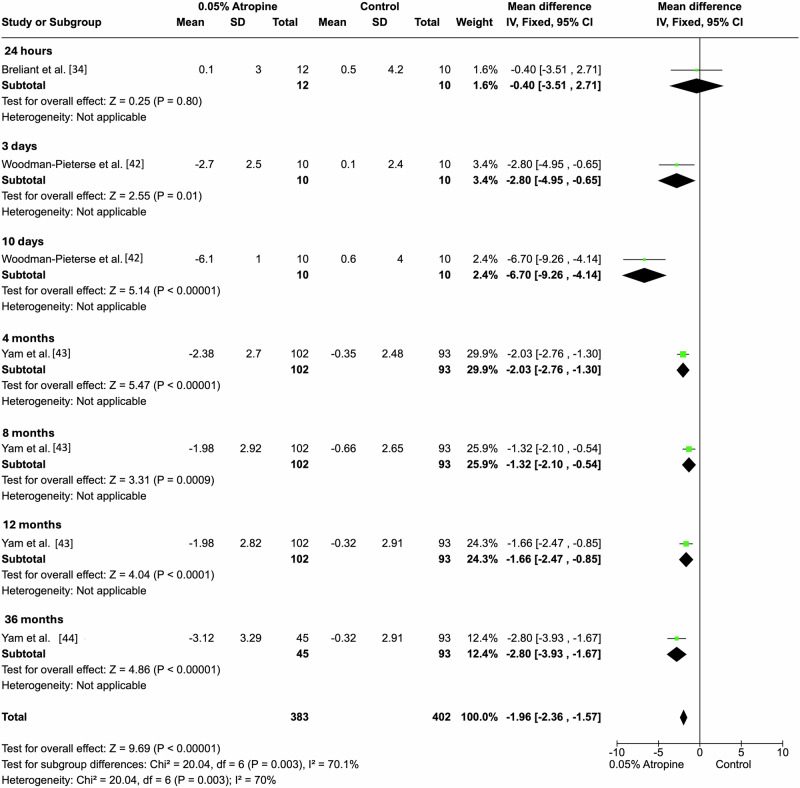
Effect of 0.05% atropine on accommodative amplitude at different follow-up periods compared with control. IV inverse variance, CI confidence interval, SD standard deviation.

Figure 4 shows the effects of 0.05% atropine on accommodative amplitude compared with controls across different follow-up periods. No significant difference was observed at 24 h [34]. In contrast, a significant reduction in accommodative amplitude was detected at very early follow-up, with marked effects at 3 and 10 days [42]. At longer follow-up durations, consistent reductions in accommodative amplitude were observed at 4, 8 and 12 months based on data from Yam et al. [43], and these effects persisted at 36 months [44]. The overall pooled analysis demonstrated a significant reduction in accommodative amplitude associated with 0.05% atropine compared with control (MD = −1.96 D, 95% CI: −2.36 to −1.57), with moderate-to-high heterogeneity across follow-up periods.

For near heterophoria, no statistically significant differences were observed between the 0.05% atropine and control groups at 24 h (MD = 1.20Δ, 95% CI: −7.72 to 10.12) or at 3 days (MD = 0.80Δ, 95% CI: −0.68 to 2.28), based on data from Breliant et al. [34] and Woodman-Pieterse et al. [42], respectively. In contrast, at 10 days, Woodman-Pieterse et al. [42] reported a significant shift in near heterophoria in the atropine group compared with the control (MD = 4.90Δ, 95% CI: 1.96–7.84). The overall pooled estimate across time points indicated a small but significant effect (MD = 1.62Δ, 95% CI: 0.31–2.92), with moderate heterogeneity.

For distance heterophoria, no significant differences were detected at any assessed follow-up. At 24 h, Breliant et al. [34] reported no effect of atropine compared with control (MD = 0.00Δ, 95% CI: −5.44 to 5.44). Similarly, no significant differences were observed at 3 days (MD = 0.00Δ, 95% CI: −1.19 to 1.19) or at 10 days (MD = −0.90Δ, 95% CI: −1.74 to 0.06) in Woodman-Pieterse et al. [42]. The overall pooled estimate confirmed the absence of a significant effect (MD = −0.59Δ, 95% CI: −1.27 to 0.09).

Regarding NFV break, no statistically significant difference was observed at 24 h [34] (MD = −4.80Δ, 95% CI: −15.50 to 5.90). At 3 days, Woodman-Pieterse et al. [42] reported a significant reduction in NFV break in the atropine group compared with control (MD = −5.90u, 95% CI: −11.33 to −0.47), which became more pronounced at 10 days (MD = −12.20Δ, 95% CI: −18.10 to −6.30). The pooled analysis across time points showed a significant reduction in NFV break associated with 0.05% atropine (MD = −8.30Δ, 95% CI: −12.05 to −4.56), with low-to-moderate heterogeneity.

For the PFV break, no significant differences were observed at any time point. At 24 h, Breliant et al. [34] reported no significant effect (MD = −4.10Δ, 95% CI: −20.66 to 12.46). Similarly, results at 3 days (MD = 5.80Δ, 95% CI: −2.06 to 13.66) and 10 days (MD = 7.20Δ, 95% CI: −2.18 to 16.58) from Woodman-Pieterse et al. [42] were not statistically significant. The overall pooled estimate confirmed the absence of a significant effect on PFV break (MD = 5.15Δ, 95% CI: −0.51 to 10.82).

#### 0.1–1% Atropine

For 0.1% atropine, a marked and statistically significant reduction in accommodative amplitude was observed at both assessed follow-up periods. At 12 months, Chia et al. [7] reported a substantial decrease in accommodative amplitude in the atropine group compared with control (MD = −10.20 D, 95% CI: −11.01 to −9.39). This effect persisted at 24 months, with a similarly large reduction (MD = −8.95 D, 95% CI: −9.83 to −8.07). The pooled analysis confirmed a strong overall effect of 0.1% atropine on accommodative amplitude (MD = −9.63 D, 95% CI: −10.22 to −9.03), indicating a clinically relevant impairment of accommodation at this concentration.

For 0.5% atropine, a pronounced reduction in accommodative amplitude was also observed. At 12 months, data from Chia et al. [7] showed a significant decrease compared with control (MD = −11.70 D, 95% CI: −12.42 to −10.98). This effect remained evident at 24 months, with a comparable reduction (MD = −10.65 D, 95% CI: −11.57 to −9.73). The overall pooled estimate demonstrated a large and statistically significant reduction in accommodative amplitude associated with 0.5% atropine (MD = −11.30 D, 95% CI: −11.87 to −10.74), suggesting a strong dose-related effect on accommodation.

In contrast, for 1% atropine, accommodative amplitude was assessed only after treatment discontinuation. At 30 months, Tong et al. [11] reported no significant difference between the atropine and control groups (MD = −1.62 D, 95% CI: −3.71 to 0.47). Similarly, at 36 months, the difference remained non-significant (MD = −0.53 D, 95% CI: −1.36 to 0.30). The pooled analysis across these time points confirmed the absence of a significant long-term difference (MD = −0.68 D, 95% CI: −1.45 to 0.09), suggesting recovery of accommodative function following cessation of treatment. Table 2 summarises the effects of atropine on accommodative amplitude during active treatment across concentrations and follow-up periods.

**Table 2:** Summary of dose- and time-dependent effects of atropine on accommodative amplitude.

Atropine concentration (%)	Overall effect on accommodative amplitude	Temporal pattern across follow-up	Interpretation
0.01	Small but statistically significant overall reduction (MD: −0.84 D)	Highly heterogeneous; not consistently significant at individual time points; significant at 18 months based on a single study	Minimal and variable accommodative impact
0.02–0.025	Small overall reduction (MD: −0.92 D)	Variable effects across follow-up periods with inconsistent significance	Transitional dose range with emerging accommodative effects
0.03	No significant short-term effect	Assessed only at 24 h in a single study	Insufficient evidence for accommodative impact
0.05	Consistent and clinically meaningful reduction (MD: −1.96 D)	Present from early follow-up (days) and persisting up to 36 months	Clinically relevant accommodative impairment
0.1	Marked reduction (MD: >−9 D)	Consistent at all assessed follow-up periods	Strong cycloplegic effect
0.5	Marked reduction (MD: >−10 D)	Consistent at all assessed follow-up periods	Strong cycloplegic effect

*MD* mean difference.

### Sensitivity analysis

A sensitivity analysis was performed to explore sources of heterogeneity in the pooled analysis of accommodative amplitude with 0.01% atropine (Additional file 4). At 6 months, exclusion of the study by Lee et al. [39] resulted in a reduction of heterogeneity from 73% to 39%, while the pooled mean difference remained non-significant. Similarly, at 12 months, removal of Chia et al. [7] led to a modest decrease in heterogeneity from 93% to 87%, with no material change in the direction or statistical significance of the pooled effect. At 24 months, exclusion of Chia et al. [7] also reduced heterogeneity substantially, from 88% to 50%, while the pooled estimate remained non-significant.

### Publication Bias

**Fig. 5:**
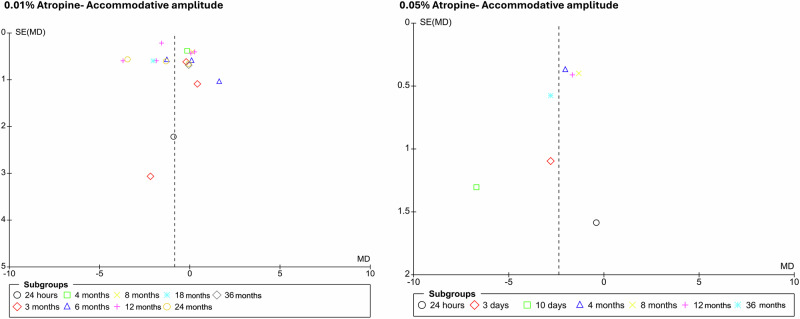
Publication bias assessment. Funnel plot assessments of publication bias for accommodative amplitude with 0.01% atropine (left panel) and 0.05% atropine (right panel). Each point represents an individual study estimate, and the dashed vertical line indicates the pooled mean difference.

Publication bias was assessed using funnel plots for accommodative amplitude with 0.01% and 0.05% atropine separately (Fig. 5). For 0.01% atropine, the funnel plot appeared largely symmetrical around the pooled effect estimate, with most studies distributed evenly on both sides of the mean difference across different follow-up periods. Although a small number of points showed greater dispersion, particularly among studies with larger standard errors, there was no clear pattern suggestive of substantial publication bias.

In contrast, the funnel plot for 0.05% atropine showed a greater spread of effect estimates, particularly at shorter follow-up periods, which may reflect increased variability and small-study effects rather than true publication bias. Despite this wider dispersion, no consistent directional asymmetry was evident. Overall, the funnel plot assessment does not indicate strong evidence of publication bias for accommodative amplitude, although interpretation should be made with caution given the limited number of studies contributing to some dose–time subgroups.

### Grade

The GRADE summary of findings for accommodative amplitude with atropine is presented in Table 3. The certainty of evidence was rated as moderate for both 0.01% atropine and 0.05% atropine compared with the control. Across the included RCTs, no serious concerns were identified regarding risk of bias, indirectness or imprecision. However, the certainty of evidence was downgraded by one level for both concentrations due to substantial heterogeneity and inconsistency of effect estimates across follow-up periods. Accommodative amplitude was classified as a critical outcome for clinical decision-making, supporting the clinical relevance of the findings despite the observed variability.

**Table 3:** Grading of Recommendations, Assessment, Development and Evaluation (GRADE) assessment of the quality of the evidence and the strength of the recommendations.

Certainty assessment							No. of participants		Effect	Certainty	Importance
No. of studies	Study design	Risk of bias	Inconsistency	Indirectness	Imprecision	Other considerations	[intervention]	[comparison]	MD (95% CI)		
*0.01% Atropine*											
11	Randomised trials	Not serious	Serious^a^	Not serious	Not serious	None	682/1420 (48.0%)	738/1420 (52.0%)	MD: −0.84 (−1.50 to −0.18)	⊕⊕⊕〇 Moderate^a^	Critical
*0.05% Atropine*											
3	Randomised trials	Not serious	Serious^b^	Not serious	Not serious	None	127/240 (52.9%)	113/240 (47.1%)	MD: −1.96 (−2.36 to −1.57)	⊕⊕⊕〇 Moderate^b^	Critical

*CI* confidence interval, *MD* mean difference, *No* number; CI: MD: ⊕ represent the strength of evidence (⊕ very low, ⊕⊕ low, ⊕⊕⊕ moderate, ⊕⊕⊕⊕ high).

^a^High heterogeneity (*I*² = 84%) with inconsistent results across follow-up periods.

^b^The certainty of evidence for the effect of 0.05% atropine on accommodative amplitude was rated as moderate, downgraded due to substantial heterogeneity (*I*² = 82%) and time-dependent variability of effect estimates.

## Discussion

The present systematic review and meta-analysis provide an integrated evaluation of the effects of atropine eye drops on accommodative amplitude and binocular visual function across a wide range of concentrations and follow-up periods. By restricting the quantitative synthesis to RCTs and stratifying outcomes by concentration and time, this work allows direct comparison with previous experimental, clinical and meta-analytic evidence, clarifying several apparent inconsistencies in the literature.

Short-term experimental studies have consistently reported marked reductions in accommodative amplitude even with very low concentrations of atropine. For example, Tran et al. [45] reported substantial reductions in accommodative amplitude after only 2 weeks of treatment with 0.01%, 0.02% and 0.03% atropine, accompanied by dose-dependent pupil dilation. Similarly, Santos-Neto et al. [46] observed significant disturbances in multiple accommodative parameters after 14 days of 0.025% and 0.05% atropine. These findings contrast with the more modest effects observed in the present meta-analysis for 0.01% atropine, where the pooled reduction in accommodative amplitude was small (MD: −0.84 D), highly heterogeneous and not consistently present at individual follow-up time points. This discrepancy likely reflects differences in study design, as short-term pre–post studies capture acute pharmacological effects without accounting for adaptation, interindividual variability or comparison with untreated controls.

Longitudinal controlled studies provide a more nuanced perspective. Li et al. [47] reported significant changes in accommodative amplitude and related parameters at 3 months with 0.01% atropine, whereas effects were attenuated or absent at later follow-up in participants without sustained accommodative demand. These findings align with the present results, which showed no consistent effect of 0.01% atropine across follow-up periods and no significant impact on stereoacuity or accommodative lag. Likewise, Cyphers et al. [48] found no clinically meaningful accommodative or binocular changes after one week of 0.01% atropine in young adults, supporting the interpretation that functional effects at this concentration are subtle and often transient.

At intermediate concentrations (0.02–0.03%), the present analysis identified a statistically significant pooled reduction in accommodative amplitude, but with substantial heterogeneity and inconsistent time-specific effects. Tran et al. [45] reported large short-term reductions at these concentrations, with a subset of eyes exhibiting residual accommodative amplitudes below 5 D, whereas the present synthesis suggests that such pronounced effects are not uniformly sustained over longer follow-up. These findings indicate that 0.02–0.03% atropine may represent a functional transition range, in which accommodative changes are more likely than with 0.01% atropine but remain variable and context dependent.

In contrast, atropine 0.05% demonstrated a consistent and clinically meaningful impact on accommodation in the present analysis, with reductions observed from early follow-up and persisting over time. For interpretative purposes, reductions in accommodative amplitude of <1 D were considered small, while reductions between 1 and 3 D and >3 D were considered as moderate and marked or clinically significant, respectively. This finding is concordant with Santos-Neto et al. [46], who reported marked reductions in accommodative amplitude, facility and relative accommodation, as well as increased pupil diameter with 0.05% concentrations. It is also supported by recent longitudinal evidence from Pan et al. [49], showing short-term changes in accommodation and binocular vision with 0.05% atropine that tended to normalise partially over 1 year. Importantly, the present meta-analysis extends these observations by providing pooled quantitative estimates and demonstrating that, unlike lower concentrations, 0.05% atropine is not only associated with reduced accommodative amplitude but also with measurable changes in binocular parameters such as near heterophoria and negative fusional vergence.

At higher concentrations (≥0.1%), the present findings confirm a strong and clinically significant reduction in accommodative amplitude, fully consistent with classic clinical trials and experimental studies. Chia et al. [7] and Lee et al. [50] demonstrated substantial cycloplegic effects with the use of 0.1–0.5% atropine, while experimental work in young adults by Lal et al. [51] showed clear dose-dependent changes in static and dynamic accommodation even after a single drop. These results reinforce the established view that moderate-to-high atropine concentrations are associated with predictable and pronounced functional impairment of accommodation.

Comparison with previous meta-analyses further highlights the contribution of the present study. Tran et al. [52] reported a nonlinear dose–response relationship between atropine concentration and changes in accommodative amplitude, with a steep increase above 0.05–0.1%. Hou et al. [53] similarly identified logarithmic dose–response relationships and suggested that concentrations ≤0.05% preserve residual accommodation above 5 D. In contrast, Wei et al. [54] concluded that atropine did not affect accommodative amplitude significantly, likely due to pooling heterogeneous study designs and follow-up periods. By restricting inclusion to RCTs and explicitly stratifying by dose and time, the present analysis reconciles these findings, demonstrating that accommodative effects are indeed dose dependent, but highly sensitive to study design, follow-up duration and outcome timing.

The findings of this study align with long-term clinical trials, indicating that atropine dosing must balance myopia-control efficacy with tolerability at near. In ATOM2, 0.01% atropine was associated with minimal accommodative loss (around 2–3 D) and little near visual impact over 5 years [12]. In contrast, higher concentrations were more likely to induce symptomatic near blur and binocular stress, which is consistent with the dose-dependent functional changes synthesised here. Additionally, evidence from the extended follow-up in the LAMP study suggests that cessation commonly necessitates restarting therapy and that re-treatment with 0.05% atropine can regain efficacy comparable to continuous treatment, supporting the need for planned monitoring of near visual function during dose escalation and when discontinuing therapy [17].

Several limitations of this systematic review and meta-analysis should be acknowledged. First, although only RCTs were included, substantial heterogeneity was observed in several pooled analyses, particularly for accommodative amplitude at low atropine concentrations. This heterogeneity likely reflects differences in atropine dosage, follow-up duration, outcome definitions and clinical methods used to assess accommodation and binocular vision across studies, as well as variability in study populations and measurement protocols. Consequently, pooled estimates should be interpreted with caution, particularly when comparing results across different atropine concentrations and follow-up periods. Second, the number of trials contributing data to some of the dose–time subgroups was limited, reducing statistical power and precluding robust exploration of moderators such as age, baseline refractive error or near-work behaviour. Third, accommodative and binocular outcomes were often secondary endpoints in the included trials and standardised measurement protocols were not applied consistently, potentially contributing to variability in effect estimates. In addition, most studies were conducted in Asian populations, which may limit the generalisability of the findings to other ethnic or geographic groups. Differences in iris pigmentation may also influence the pharmacological response to atropine, including its effects on accommodation. However, this variable was not reported consistently across the included studies, and therefore may limit the generalisability of the findings, particularly given that most trials were conducted in Asian populations. Thus, future research should prioritise large, well-designed RCTs with standardised and clinically relevant assessment of accommodative and binocular visual function as prespecified primary outcomes. Long-term studies examining functional adaptation over extended treatment durations and after treatment cessation are particularly needed. Furthermore, investigations comparing different dosing regimens and frequencies, as well as exploring interactions between atropine therapy, near-work behaviour and optical interventions, may help optimise individualised myopia management strategies.

## Conclusions

This systematic review and meta-analysis demonstrates that the effects of atropine eye drops on accommodative amplitude and binocular visual function in myopic children are clearly dose-dependent and time sensitive. Low-dose atropine (0.01%) is associated with only a small and inconsistent reduction in accommodative amplitude, with no significant effects on accommodative lag, stereoacuity or most binocular vision parameters, supporting its favourable functional safety profile. Intermediate concentrations (0.02–0.03%) show greater variability, suggesting a transition range in which accommodative effects may emerge in a subset of patients. In contrast, 0.05% atropine consistently produces clinically meaningful reductions in accommodative amplitude and measurable changes in binocular function, while higher concentrations (≥0.1%) result in marked and predictable cycloplegic effects.

By synthesising randomised controlled evidence and stratifying outcomes by dose and follow-up duration, this review clarifies discrepancies in previous studies and highlights the importance of balancing myopia control efficacy with preservation of visual function. The findings provide clinically relevant guidance for evidence-based atropine dosing in paediatric myopia management.

## Supplementary information

Additional File 1

Additional File 2

Additional file 3

Additional file 4

## Data Availability

No datasets were generated or analysed during the current study.
