# Supplementary material for: Dose-Dependent Effects of Atropine on Accommodative and Binocular Visual Function for Myopia Control in Children: A Systematic Review and Meta-Analysis
Source: Ophthalmic Physiol Opt. 2026 May 18;46(3):681–94. doi: 10.1007/s44402-026-00093-5 (PMC13369229; doi:10.1007/s44402-026-00093-5)
Supplement: Supplementary file 2 — Additional File 2 [file 44402_2026_93_MOESM2_ESM.docx]

**Additional File 2.** Full electronic search strategies.

Pubmed
Search: 
Search: **(atropine[MeSH Terms] OR atropine[Title/Abstract]) AND (child*[Title/Abstract] OR pediatric*[Title/Abstract] OR paediatric*[Title/Abstract] OR adolescent*[Title/Abstract]) AND (accommodation[Title/Abstract] OR accommodative[Title/Abstract] OR "accommodative amplitude"[Title/Abstract] OR "accommodative lag"[Title/Abstract] OR "binocular vision"[Title/Abstract] OR binocular[Title/Abstract] OR vergence[Title/Abstract] OR "near point of convergence"[Title/Abstract] OR NPC[Title/Abstract] OR phoria[Title/Abstract] OR exophoria[Title/Abstract] OR esophoria[Title/Abstract] OR "AC/A"[Title/Abstract] OR "convergence insufficiency"[Title/Abstract]) OR "accommodative function"[Title/Abstract]** Sort by: **Publication Date**

(("hyoscyamine"[MeSH Terms] OR "atropine"[MeSH Terms] OR "atropine"[Title/Abstract]) AND ("child*"[Title/Abstract] OR "pediatric*"[Title/Abstract] OR "paediatric*"[Title/Abstract] OR "adolescent*"[Title/Abstract]) AND ("accommodation"[Title/Abstract] OR "accommodative"[Title/Abstract] OR "accommodative amplitude"[Title/Abstract] OR "accommodative lag"[Title/Abstract] OR "binocular vision"[Title/Abstract] OR "binocular"[Title/Abstract] OR "vergence"[Title/Abstract] OR "near point of convergence"[Title/Abstract] OR "NPC"[Title/Abstract] OR "phoria"[Title/Abstract] OR "exophoria"[Title/Abstract] OR "esophoria"[Title/Abstract] OR "AC/A"[Title/Abstract] OR "convergence insufficiency"[Title/Abstract])) OR "accommodative function"[Title/Abstract]

**Translations**

**atropine[MeSH Terms]:** "hyoscyamine"[MeSH Terms] OR "atropine"[MeSH Terms]

Web of Science

((TS=(atropine OR atropine[MeSH Terms])) AND

(TS=(child* OR pediatric* OR paediatric* OR adolescent*)) AND

(TS=(accommodation OR accommodative OR "accommodative amplitude" OR "accommodative lag" OR "binocular vision" OR binocular OR vergence OR "near point of convergence" OR NPC OR phoria OR exophoria OR esophoria OR "AC/A" OR "convergence insufficiency" OR "accommodative function")))

Scopus
(TITLE-ABS-KEY(atropine) OR TITLE-ABS-KEY("atropine[MeSH Terms]")) AND

(TITLE-ABS-KEY(child* OR pediatric* OR paediatric* OR adolescent*)) AND

(TITLE-ABS-KEY(accommodation OR accommodative OR "accommodative amplitude" OR "accommodative lag" OR "binocular vision" OR binocular OR vergence OR "near point of convergence" OR NPC OR phoria OR exophoria OR esophoria OR "AC/A" OR "convergence insufficiency" OR "accommodative function"))
