# Supplementary material for: Dose-Dependent Effects of Atropine on Accommodative and Binocular Visual Function for Myopia Control in Children: A Systematic Review and Meta-Analysis
Source: Ophthalmic Physiol Opt. 2026 May 18;46(3):681–94. doi: 10.1007/s44402-026-00093-5 (PMC13369229; doi:10.1007/s44402-026-00093-5)
Supplement: Supplementary file 3 — Additional file 3 [file 44402_2026_93_MOESM3_ESM.docx]

**Additional file 3.** Risk of bias judgement.

Breliant et al. (2023)

A) Participants were randomized into four groups (placebo, 0.01%, 0.03%, and 0.05% atropine). The randomization sequence was generated using Microsoft Excel with the aim of achieving equivalent baseline age and refractive error across groups.

B) Although participants were randomized, the method used to conceal allocation prior to assignment was not described. There is no information regarding whether allocation was concealed from investigators enrolling participants.

C) The study was described as a randomized double-masked trial. Participants received either atropine or placebo eye drops prepared by a compounding pharmacy, with identical components except for atropine, indicating adequate masking of participants and study personnel.

D) Outcome assessors were masked as part of the double-masked study design. Objective and standardized measurements (e.g., pupillometry, autorefractor measurements) further reduce the likelihood of detection bias.

E) All 46 randomized participants were included in the analysis, with no reported loss to follow-up or missing outcome data across the study time points (baseline, 30 minutes, 60 minutes, and 24 hours).

F) All outcomes described in the Methods section (visual acuity, pupil size, binocular vision parameters, and accommodation measures) were fully reported in the Results section, with no evidence of selective outcome reporting.

H) The study had a relatively small sample size and short follow-up period. In addition, atropine concentrations were not independently verified, and randomization did not account for iris color or race/ethnicity, which may influence pupil response. However, no clear evidence of additional bias was identified.

Chia et al. (2012)

A) Children were randomly assigned to the 0.5%, 0.1%, and 0.01% atropine groups in a predefined 2:2:1 ratio as part of a randomized study design.

B) The study reports random assignment and double masking, but the method used to conceal allocation prior to participant enrollment was not described.

C) The trial was described as double-masked. Participants received atropine eye drops at different concentrations administered nightly, with masking intended to prevent participants and personnel from knowing treatment allocation.

D) Outcome assessors were masked as part of the double-masked design. Primary outcomes such as cycloplegic refraction and axial length are objective measurements, reducing the risk of detection bias.

E) The study followed participants for 2 years with scheduled follow-up visits. Outcome data were reported for all treatment groups, and no evidence of differential attrition affecting outcomes was reported.

F) Primary and secondary outcomes prespecified in the Methods section, including myopia progression, axial length, accommodation, pupil size, and visual acuity, were all reported in the Results.

H) The study was a large, well-powered randomized clinical trial with standardized outcome measurements and balanced baseline characteristics. No additional sources of bias were identified.

Cui et al. (2021)

A) Eligible participants were randomly assigned to treatment groups. Subjects receiving atropine were double-blind randomized to either 0.01% or 0.02% atropine, indicating the use of a random sequence generation process.

B) Although randomization between the two atropine concentrations was described as double-blinded, the method used to conceal allocation was not reported. In addition, assignment to atropine versus control was not randomized.

C) Participants were not fully blinded to treatment allocation because the control group did not receive placebo eye drops and only wore spectacles. Therefore, masking of participants and personnel was not possible for comparisons involving the control group.

D) Outcome measurements such as cycloplegic autorefraction and axial length were obtained using standardized, objective instruments. Examiners were masked to atropine concentration, reducing the risk of detection bias.

E) Attrition was reported and reasons for withdrawal were provided. Baseline characteristics of participants who completed the study and those lost to follow-up were similar, suggesting that missing data were unlikely to bias the results.

F) All prespecified outcomes described in the Methods section, including spherical equivalent refractive error, axial length, pupil diameter, and accommodation amplitude, were fully reported.

H) Participants were allowed to choose atropine treatment versus no atropine due to ethical requirements, resulting in a non-randomized control group. This introduces potential selection and expectation bias beyond standard randomization domains.

Fu et al. (2020)

A) Children assigned to atropine treatment were randomly allocated in a double-blinded manner to receive either 0.01% or 0.02% atropine eye drops.

B) Although randomisation between atropine concentrations was described as double-blinded, the method used to conceal allocation was not reported. In addition, allocation to atropine versus control was not randomised.

C) Participants in the control group did not receive placebo eye drops and therefore were aware of not receiving atropine. Full masking of participants and personnel was not possible for comparisons involving the control group.

D) All outcome measurements were performed by the same clinician masked to atropine concentration, and primary outcomes such as spherical equivalent refraction and axial length were obtained using objective, standardised instruments.

E) Attrition rates were similar across groups and reasons for dropout were reported. Baseline characteristics of participants who completed the study and those lost to follow-up were comparable.

F) All outcomes prespecified in the Methods section, including myopia progression, axial length, pupil diameter, accommodative amplitude and adverse effects, were fully reported.

H) Participants were allowed to choose atropine treatment versus no atropine due to ethical requirements, resulting in a non-randomised control group without placebo. This introduces potential selection and expectation bias.

Hvid-Hansen et al. (2023)

A) Participants were randomized using a computer-generated randomization list with variable block sizes and stratification by study site. The randomization list was generated by an independent researcher prior to study initiation.

B) Allocation concealment was ensured by the use of sealed randomization codes handled by an independent researcher and a compounding pharmacy. Investigators, participants, and outcome assessors were masked to treatment allocation throughout the study.

C) The study was double-masked and placebo-controlled. Participants, parents, investigators, and clinical staff were unaware of treatment allocation. Placebo eye drops were identical in appearance and administration to active treatment.

D) Outcome assessors were masked to treatment allocation. Primary outcomes, including axial length and cycloplegic spherical equivalent refraction, were obtained using objective, standardized instruments.

E) Attrition during the first six months was minimal, with only one participant withdrawing. Missing data were handled using maximum likelihood estimation in linear mixed models.

F) The study protocol was registered prior to initiation, and all prespecified outcomes were reported in the published manuscript.

H) The study was investigator-initiated, multicenter, conducted under GCP monitoring, and funded by independent research foundations. No conflicts of interest were declared, and baseline characteristics were balanced across groups.

Jiang et al. (2023)

A) Participants were randomly allocated in a 1:1:1:1 ratio to four intervention groups, as described in this prospective randomized controlled clinical trial.

B) Although participants were randomized and atropine and placebo solutions were packaged with similar appearance and labeling, the method used to conceal allocation during enrolment was not described.

C) Masking of participants and personnel was not possible across all groups due to the nature of the interventions, particularly orthokeratology lens wear versus spectacle correction.

D) All measurements were performed by the same experienced optometrists using standardized procedures; however, masking of outcome assessors to group allocation was not explicitly stated.

E) Attrition was minimal, with only two participants lost to follow-up, and reasons for withdrawal were reported. Outcome data were available for the majority of participants.

F) All binocular vision outcomes described in the Methods section were reported in the Results, with no evidence of selective outcome reporting.

H) Binocular vision measurements were conducted in a small predefined subgroup of the original randomized cohort, resulting in limited sample size and reduced statistical power. In addition, the short follow-up period (3 months) limits inference on long-term effects.

Lee et al. (2022)

A) Participants were allocated to the treatment or placebo group using a simple randomisation process in this randomised clinical trial.

B) Placebo and atropine eyedrops were packaged identically and labelled only with participant details, preventing identification of group allocation at enrolment.

C) This was a double-masked, placebo-controlled trial with identical treatment and placebo formulations.

D) Outcome measures were collected using objective instruments, and masking was maintained throughout the trial.

E) Differential dropout was observed between groups, with significantly higher withdrawal in the placebo group at 18 and 24 months.

F) All primary and secondary outcomes pre-specified in the protocol were reported.

H) Baseline differences in age and ocular biometric parameters were present between groups, although these were adjusted for in the statistical models.

Saxena et al. (2021)

A) Participants were randomized in a 1:1 ratio using computer-generated random numbers.

B) Bottles were similarly labeled with subject identification numbers, and investigators and participants were masked to the type of intervention.

C) This was a double-masked, placebo-controlled randomized clinical trial.

D) Outcome assessments were performed by optometrists masked to group allocation using objective instruments.

E) Only 8 out of 100 participants were lost to follow-up, with balanced attrition between groups.

F) Primary and secondary outcomes were clearly defined and fully reported.

H) Baseline demographic and ocular parameters were comparable between groups, and the sponsor had no role in study conduct or analysis.

Tong et al. (2009)

A) Assignments to treatment were allocated with concealment according to a computer-generated randomization list.

B) Randomization assignments were concealed; study medication was dispensed immediately after randomization in identical bottles.

C) This was a double-masked, placebo-controlled trial with identical packaging of atropine and placebo eyedrops.

D) Investigators measuring outcomes were masked, and both pupils were dilated before assessment to preserve masking.

E) 333 of 400 children completed the third-year follow-up; dropout rates were similar between groups and unrelated to outcomes.

F) Prespecified primary and secondary outcomes (SE, axial length, accommodation) were fully reported.

H) Baseline characteristics were comparable; no commercial conflicts of interest were declared.

Wang et al. (2020)

A) Participants were randomized to receive atropine 0.01% or single-vision lenses.

B) No explicit description of allocation concealment procedures.

C) Examiners were masked to group allocation.

D) Examinations were conducted by study-certified examiners masked to the group allocation.

E) 61 of 63 participants completed the 6-month follow-up; one participant in each group withdrew.

F) Prespecified primary and secondary outcomes were fully reported.

H) Early termination recommended by Data and Safety Monitoring Committee due to faster progression in the control group.

Woodman-Pieterse et al. (2025)

A) Participants were allocated to the treatment or control group (1:1 ratio) via a block randomisation scheme.

B) Eye drops were labelled as ‘Solution A’ and ‘Solution B’ and dispensed by an independent compounding pharmacy.

C) This was a randomised, double-masked clinical trial; however, mydriasis and cycloplegia were apparent in some participants from day 3.

D) Data collection was masked; however, several outcomes required subjective participant responses.

E) One participant withdrew due to an unrelated adverse event prior to drop use; all remaining participants completed follow-up.

F) All prespecified accommodation and vergence outcomes were fully reported.

H) Adult population (18–25 years), short duration (10 days), small sample size (n=20).

Yam et al. (2019)

A) Participants were randomized in a 1:1:1:1 ratio in 6 strata defined by gender and age groups.

B) Trial medications were prepackaged identically and labeled only with subject numbers and expiration dates.

C) Parents, subjects, and investigators were masked to trial medications.

D) All ophthalmic parameters were assessed by masked optometrists following standardized protocols.

E) Attrition rates were low and balanced across groups (6.4–16.2%), and analyses were performed using intention-to-treat.

F) All prespecified primary and secondary outcomes were fully reported, including safety and quality-of-life measures.

H) Placebo group was limited to 1 year due to ethical considerations, and accommodation was measured using the RAF rule.

Yam et al. (2021)

A) At the beginning of the third year, children in each group were randomized at a 1:1 ratio to continued treatment and washout subgroups, stratified by sex and age.

B) Trial medication was prepackaged as mono-dose eye drops; investigators remained masked to all group and subgroup allocations.

C) All study subjects and their parents were informed about whether they were assigned to a continued treatment subgroup or a washout subgroup.

D) Clinical investigators remained masked to all group and subgroup allocations; outcomes were assessed using objective instruments.

E) Dropout rates over 3 years ranged from 17.4% to 35.1%, higher in the switchover group and in older subjects.

F) All prespecified primary and secondary outcomes, including rebound effects, accommodation amplitude, pupil size and quality of life, were fully reported.

H) Placebo group was switched to active treatment after year 1 for ethical reasons; washout groups were not given placebo.
