# Supplementary material for: Dose-Dependent Effects of Atropine on Accommodative and Binocular Visual Function for Myopia Control in Children: A Systematic Review and Meta-Analysis
Source: Ophthalmic Physiol Opt. 2026 May 18;46(3):681–94. doi: 10.1007/s44402-026-00093-5 (PMC13369229; doi:10.1007/s44402-026-00093-5)

**Additional file 4**. Sensitivity analysis of the effect of 0.01% atropine on accommodative amplitude across follow-up periods.


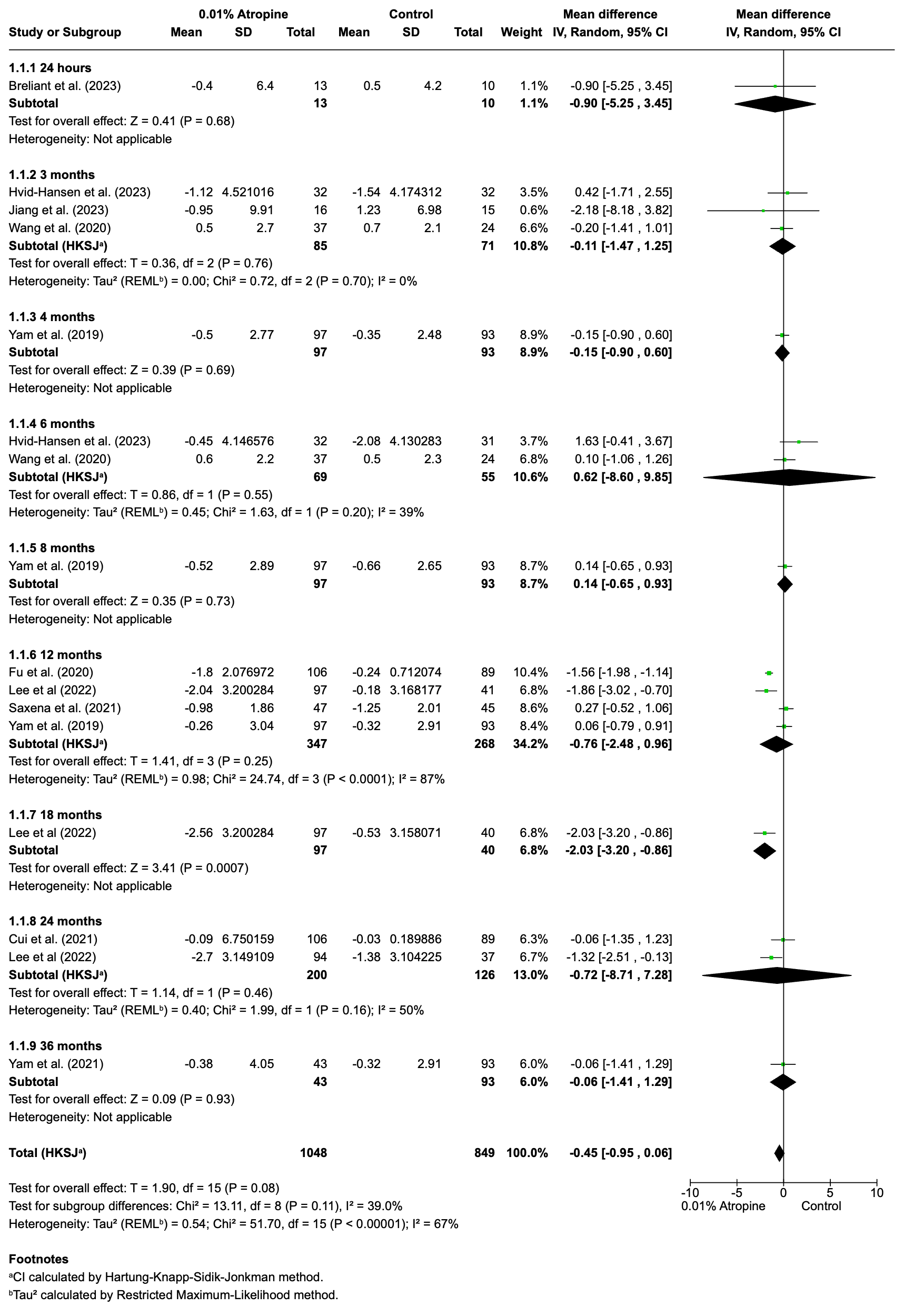

Supplement: Supplementary file 4 — Additional file 4 [file 44402_2026_93_MOESM4_ESM.docx]
